# MPPE/SEBS Composites with Low Dielectric Loss for High-Frequency Copper Clad Laminates Applications

**DOI:** 10.3390/polym12091875

**Published:** 2020-08-20

**Authors:** Jianming Guo, Hao Wang, Caixia Zhang, Qilong Zhang, Hui Yang

**Affiliations:** 1 School of Materials Science and Engineering, State Key Lab Silicon Mat, Zhejiang University, Hangzhou 310027, China; 21826022@zju.edu.cn (J.G.); ustbwanghao@163.com (H.W.); yanghui@zju.edu.cn (H.Y.); 2 Jiaxing Glead Elect Co Ltd., Jiaxing 314003, China; z363238730@163.com

**Keywords:** polymer composites, low dielectric loss, processability, thermal properties

## Abstract

Copper clad laminates (CCLs) with low dissipation factor (Df) are urgently needed in the fields of high-frequency communications devices. A novel resin matrix of modified poly (2,6-dimethyl-1,4-phenylene ether) (MPPE) and styrene-ethylene/butylene-styrene (SEBS) was employed in the fabrication of high-frequency copper clad laminates (CCLs). The composites were reinforced by E-glass fabrics, which were modified with phenyltriethoxysilane (PhTES). The composite laminates obtained exhibited impressive dielectric loss of 0.0027 at 10 GHz when the weight ratio of MPPE to SEBS was 5:1. In order to modify the dielectric constant (Dk), coefficient of thermal expansion (CTE) and other performances of laminates, Li_2_TiO_3_ (LT) ceramic powders were introduced into the resin matrix. The composite laminates showed low dielectric loss of 0.0026 at 10 GHz and relatively high flexural strength of 125 MPa when the mass ratio of LT fillers to resin is 0.4. Moreover, the composite laminates all maintain low water uptake (<0.5%). The microstructure and thermal properties of composite laminates filled with LT ceramic powders were also tested. These results show that copper clad laminates prepared with modified polyphenylene ether (MPPE)/SEBS and LT ceramic fillers have strong competitiveness to fabricate printed circuit boards (PCBs) for high-frequency and high-speed applications.

## 1. Introduction

With the rapid development of electronic technology and the advent of the 5G era, unprecedented opportunities have been brought to the research and development of high-performance printed circuit boards (PCBs) worldwide. High-frequency and high-speed PCBs are needed in the fields of microwave components, automotive electronics, satellite broadcasting and communication, radar and other high-frequency communications devices. As the important raw material of PCBs, copper clad laminates (CCLs) serve as a basic structural foundation and connect the loaded electronic components. Since the high-performance PCBs transfer information in high frequency and high speed, CCLs with low dissipation factor (Df) are getting increasing attentions and researches. The pursuit of light and high speed of electronic products has accordingly driven the development of CCLs to the direction of high integration, miniaturization, and multilayer [1]. The CCLs consist of insulation layer and copper foils, and the performances of CCLs are mainly determined by the insulation layer. The insulation layer of CCLs is polymeric composite, which is made of polymer resins, ceramic powders, and glass fabrics. Therefore, the polymeric composites with a low dissipation factor (Df) is ideal for application in high-frequency copper clad laminates.

Most of polymer resins used in traditional low-frequency CCLs are epoxy and phenolic, which are hard to meet the demands of low dissipation factor in high-frequency and high-speed application field [2]. The dielectric loss of polymer is connected with the polarity of polymer molecules, the density of polar groups, and the mobility of polar groups [3]. Up to now, numerous polymeric composites are synthesized and analyzed for the potential application in electronics devices. The resin matrices including modified epoxy resins, polytetrafluoroethylene (PTFE), cyanate ester (CE), polyimide resin (PI), polyphenylene ether (PPE), and other hydrocarbon resins [3,4,5,6,7]. Despite modified epoxy resins, CE and PI show relatively good dielectric and thermal properties, they still cannot meet the requirement of ultra-low dielectric loss (<0.003) at high-frequency. PTFE, PPE, and partial hydrocarbon resins can meet this requirement for their low polarizability. Among these resins, PTFE shows the lowest Df of 0.0003 (at 10 GHz) as well as extremely low water absorption by its special structure. However, PTFE is a high-temperature thermoplastic resin, which is not conducive to the manufacture of multi-layer plates. Besides, PTFE-based composites substrates need high processing temperature (>330 °C), which makes high requirements for equipment and consumes energy largely. Therefore, PPE-based resin and other hydrocarbon resins are gradually used in the manufacture of high-performance dielectric materials.

**Figure 1:**
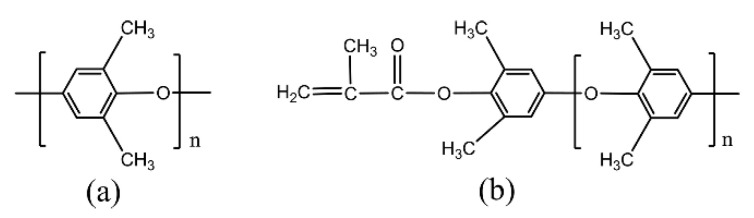
Chemical structures of (**a**) polyphenylene ether (PPE) and (**b**) modified PPE (MPPE).

Polyphenylene ether (PPE) is a thermoplastics resin with high glass transition temperature (225 °C) and low dielectric loss of 0.0007, which has great application potential in the manufacture of high-frequency copper clad laminates [8]. Many efforts have been made on the study of PPE-modified other resins to prepare composite laminates [9,10,11]. However, the high molecular weight and the poor miscibility of PPE lead to high melting temperature and high viscosity, which greatly restrict the use of PPE. Meanwhile, the phase separation can be caused by the poor compatibility between PPE and other resins. The method to overcome these drawbacks is introducing active groups into the polymer, which can change the PPE from thermoplastic to thermosetting. Herein, we use the modified PPE (MPPE) by introducing active allyl groups into PPE (Figure 1). In addition, the hydrocarbon resin styrene-ethylene/butylene-styrene (SEBS) block copolymers were also used as the resin matrix. SEBS have excellent miscibility, dielectric properties, and low water absorption. Structurally, the PS segment of SEBS has good compatibility with phenyl groups of MPPE, making it an appropriate toughened agent to MPPE. Gupta et al. [12] studied the tensile yield behavior of PP/SEBS blends. Their works stated that SEBS can improve the impact resistance, the work of yield, and processability of PP. Furthermore, they found solution blending produces can lower the discontinuity of the two-phase blend.

In order to decrease dielectric loss and modify the dielectric constant (Dk) as well as coefficient of thermal expansion (CTE) of polymeric composites, different inorganic fillers were added into the resin matrix [13,14,15]. In the light of our previous works, Li_2_TiO_3_-MgO-LiF (LT) ceramic is an extremely good additive with Dk of 23.2, Df of 0.00012 at 8 GHz [16]. However, the incompatibility of resin, ceramic powders and fiber fabrics can lead to the interface defects and the unexpectable agglomeration of ceramic powders, which could increase the dielectric loss. Herein, different silane coupling agents (SCAs) have been applied to improve the interface between resins and inorganic fillers. Meanwhile, these surface modification agents can promote the homogeneous distribution of fillers and reduce the water absorption of composites [17,18,19].

In this paper, modified polyphenylene ether (MPPE) and SEBS were used as resin matrix and the LT ceramic particles of different weight fractions were added into resin glue for the preparation of composite laminates. Meanwhile, the E-glass fabrics and synthesized LT particles were modified by Phenyltriethoxysilane (PhTES) and KH570 respectively to improve adhesive force as well as reducing the water absorption. The dielectric, thermal, mechanical, and other properties of laminates with different weight ratio of MPPE, SEBS, and Li_2_TiO_3_ ceramic particles were systematically studied.

## 2. Materials and Methods

### 2.1. Materials

Modified poly (2,6-dimethyl-1,4-phenylene ether) (MPPE) and SEBS used herein were purchased from Kraton Co., Ltd. (Dover, OH, USA). The E-glass fabrics were purchased from CPIC Co., Ltd. (Chongqing, China). Phenyltriethoxysilane (PhTES) and KH570 were obtained from Shanghai Darui Fine Chemicals Co., Ltd. (Shanghai, China). The resin was initiated by dibutylperoxide (DTBP), which is purchased from TCI (Shanghai, China). TiO_2_ (99.9%), Li_2_CO_3_ (99.9%), LiF (99.0%), MgO (99.0%), and other solvents were purchased from Aldrich (Shanghai, China).

### 2.2. Synthesis and Surface Modification of LT Powders and E-Glass Fabrics

LT ceramic powders were synthesized by the conventional solid-state route according to our previous works [16]. Coupling agents phenyltriethoxysilane (PhTES) and KH570 were used respectively to modify the surfaces of E-glass fabrics and LT powders. The E-glass fabrics were cut into fixed size and socked in the deionized water and dried at 80 °C for 6 h. The PhTES was hydrolyzed with ethanol, deionized water and a small amount of diluted hydrochloric acid to maintain a weak acid environment at 60 °C for 1 h. After the hydrolysis, immersing the E-glass fabrics in the solution and ultrasonic dispersing for 1 h, then the E-glass fabrics are dried at 120 °C for 8 h. Likewise, the surface of LT powders was modified with KH570 and then the particles were dried and ground.

### 2.3. Preparation Process of Composite Laminates

**Figure 2:**
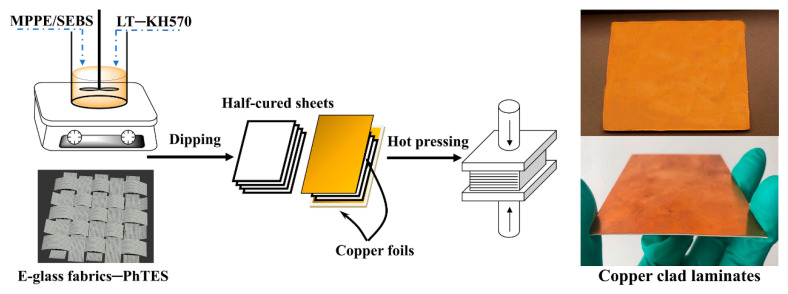
Technological process of copper clad laminates (CCLs) and the real products of CCLs-based MPPE. The CCLs were prepared with four layers of 2116 E-glass fabrics between two layers of 35 μm copper foils via hot pressing.

The fabrication procedure of MPPE-based CCLs is depicted in Figure 2. First, we fabricated CCLs without adding LT powders to determine the best weight ratio of MPPE and SEBS. In this experiment, MPPE and SEBS were added into xylene and the mixture was stirred to obtain a homogeneous solution. The weight ratios of MPPE to SEBS were 10:1, 8:1, 6:1, 5:1, 4:1, and 3:1. Then the initiator DTBP (4 wt %; mass ratio of DTBP to MPPE) was added, and the resin glue solution was stirred at 60 °C for 6 h. Subsequently, some resin glue solutions were mixed with different content of LT powders. Both of these two solutions were used to prepare composite laminates. Then the E-glass fabrics were coated and dipped with these two solutions and dried in 80 °C for 4 h to remove the solvents, and the half-cured sheets was obtained.

The 4-layers half-cured sheets were stacked together and placed between copper foil (0.035 mm) during hot pressing. Suitable hot-pressing pressure and temperature were applied to make the thickness of composite laminates at 0.50 mm. The following performance tests use the insulation layers without the coppers foils. For simplicity, some half-cured sheets were hot-pressed without the copper foil. The composite laminates obtained with resins and E-glass fabrics are abbreviated as P-CCLs. The dielectric, mechanical, and other properties of P-CCLs were analyzed before adding LT ceramic powders and the best weight ratio of MPPE to SEBS is 5:1. Similarly, the composite laminates (MPPE: SEBS = 5:1) filled with LT ceramic powders were abbreviated as LT-CCLs.

### 2.4. Characterization

The microstructures of ceramic particles and CCLs were observed from a scanning electron microscopy (FESEM, SU8010, Hitachi Ltd., Tokyo, Japan). The crystal structure of ceramic powders is analyzed by X-ray diffractometer (XRD, EMPYREAN, PANalytical Co., Almelo, The Netherlands) with Cu Kα radiation. The particle size distribution of LT ceramic powders was measured by laser particle size analyzer (Beckman Coulter LS-230 Coulter, Brea, CA, USA). The contact angle measuring device (OCA 20, Dataphysics Co., Stuttgart, Germany) was applied to measure the contact angle of the modified E-glass fabrics and LT ceramic powders. Differential scanning calorimeter (DSC) analyses were recorded with TA Q200 (TA Instruments, New Castle, DE, USA) in the nitrogen flow, through the temperature range of 20–200 °C at a heating rate of 10 °C/min. The functional groups were detected by means of Fourier transform infrared spectra (FTIR) and carried out on a NICOLET 5700 spectrometer (Thermo Electron Co., Waltham, WA, USA) at room temperature.

The coppers foils were etched according to TM-650 2.3.7A in ferric chloride solution. The dielectric properties of CCLs were tested with Split-Cylinder Resonator (QWED) at 10 GHz. TMAQ400EM was used to measure the thermal expansion in the Z-Axis according to IPC-TM-650 2.4.24. 

The flexural strength was measured by universal materials testing machine (CMT5205) according to IPC-TM-650 2.4.4B.

F = 3P × L⁄2d × h^2^ (1)

where F is the flexural strength of insulation layer, N/mm; P is the load at breaking, N; L is the span, mm; d is the width of specimens, mm; h is the thickness of the insulation layer, mm.

The water absorption was tested with IPC-TM-650 2.6.2.1 standard and CCLs were cut into 2.0 inches by 2.0 inches samples after etched. The edges of the specimens should be milled with sandpaper before test. Then the specimens were put into a container of distilled water at 23 ± 1.1 °C for 24 h. The surface water was removed with a dry cloth and all specimens should be weighed quickly.

## 3. Result and Discussion

### 3.1. Microstructure Analysis

**Figure 3:**
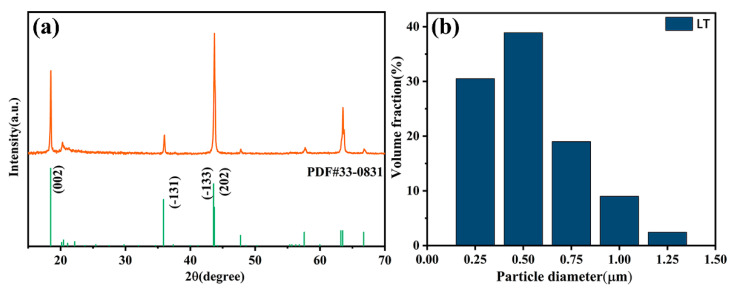
(**a**) XRD pattern of Li_2_TiO_3_-MgO-LiF (LT) powders; (**b**) particle diameter distribution of LT powders after ball milling.

The XRD patterns of Li_2_TiO_3_ powders are shown in Figure 3a. Compared with the standard data of JCPDS No. 33–0831, the peak positions are precisely matched and the pure Li_2_TiO_3_ phase is confirmed. Figure 3b depicts the particle diameter of LT powders after ball-milling and surface modification are well-distributed in the range of 0.25–1.25 μm. The mean particle diameter of LT ceramic filler is 0.42 μm, which indicates that the particle size is well controlled.

**Figure 4:**
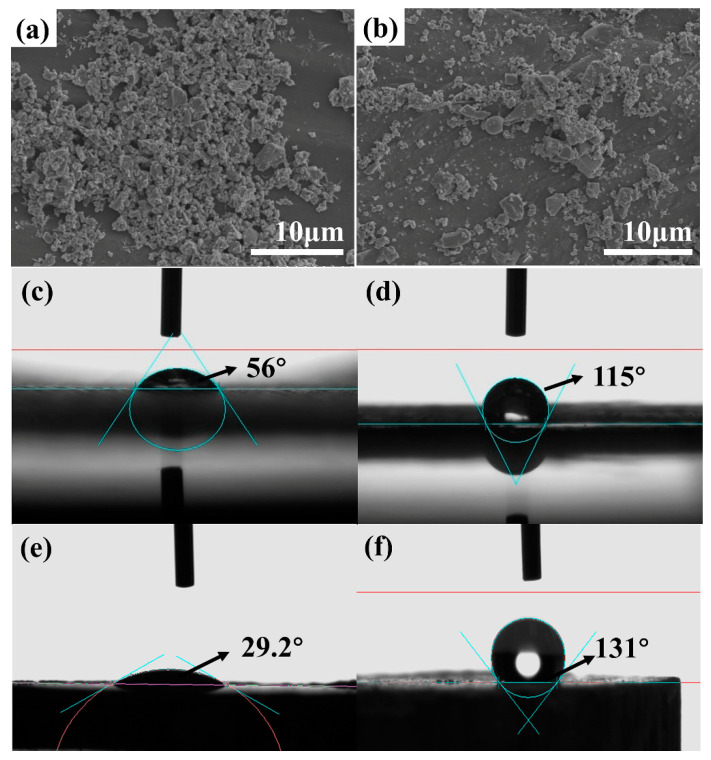
SEM images of (**a**) unmodified LT powders and (**b**) LT-KH570; (**c**,**d**) contact angle of E-glass before and after modification; (**e**,**f**) contact angle of LT powders before and after modification.

Figure 4a,b shows the SEM images of untreated LT powders and LT-KH570. Since the LT ceramic filler was ball-milled with ethanol medium, a large amount of surface hydroxyl can exist after ball milling. The surface hydroxyl is a hydrophilic group and causes the agglomeration of the raw LT powders in Figure 4a. However, Figure 4b shows that the modified powders are well-distributed. In other words, the coupling agent decreases the interaction among LT powders, and improve the compatibility of filler and polymer [14,20]. The water contact angle is measured and shown in Figure 4c–f. The contact angle of untreated E-glass fabrics is 56.1°, while it becomes 115° after PhTES modification. Meanwhile, the contact angle of LT powders improves from 29.2° to 131° after KH570 modification. Both of them change from hydrophilic to hydrophobic, which can promote the adhesion of resin and E-glass fabrics and improve the compatibility of LT powders and the resin.

**Figure 5:**
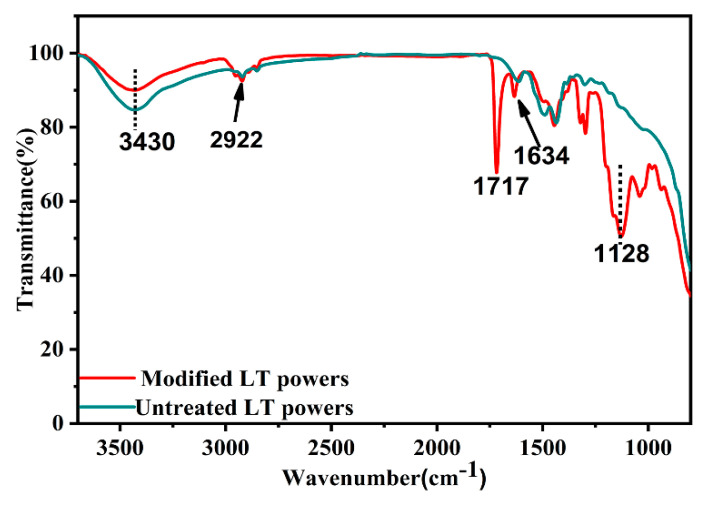
FTIR spectrum of untreated and modified LT powders.

Figure 5 shows the FTIR spectra of LT powders before and after modification. The stretching vibration of -OH group at 3430 cm^−1^ can both be found and the unmodified LT powders surface have higher intensity. Compared with unmodified LT powders, new peaks appeared at 2922 cm^−1^,1717 cm^−1^, 1634 cm^−1^, and 1128 cm^−1^ on the FTIR of spectrum of KH570-LT, which indicated the vibration peak of C–H, C=O, C=C, and C–O respectively [18,21]. The results indicated that KH570 was chemically grafted on the surface of LT particles, which can improve the dispersion and hydrophobicity of LT powders.

**Figure 6:**
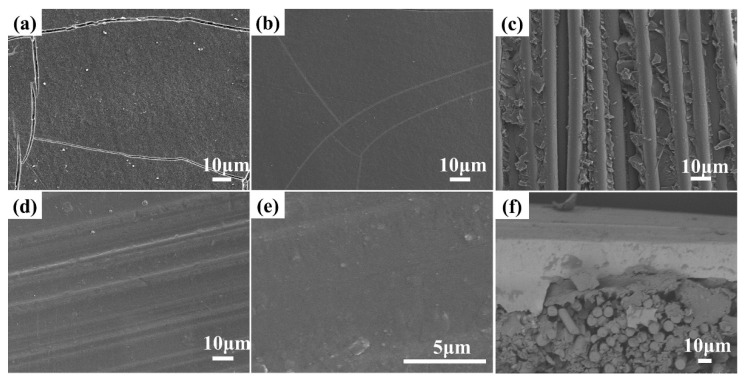
Surface SEM images of (**a**,**b**) insulation layers of P-CCLs; (**c**) filler and resin on the E-glass fabric; (**d**,**e**) insulation layers of LT-CCLs; (**f**) the cross sectional SEM images of LT-CCLs.

**Figure 7:**
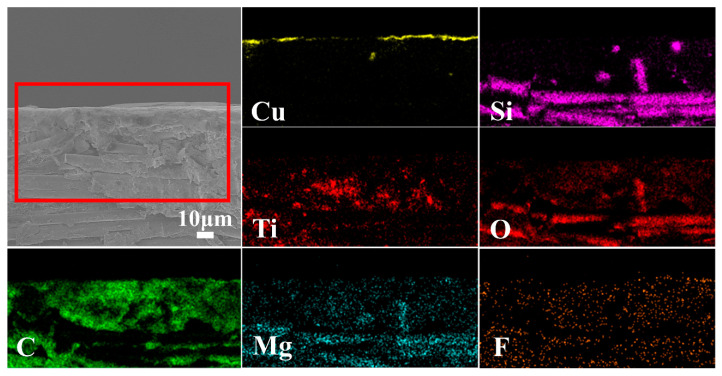
Corresponding element mapping of Cu, Si, Ti, O, C, Mg, F of LT-CCLs.

The surface SEM micrographs of insulation layers of P-CCLs and LT-CCLs are shown in Figure 6a,b,d,e respectively. As graphs show, the surface morphology has been improved with the adding of LT powders. The cracks on the surface of P-CCLs may arise from the poor flowability of the resin matrix during the hot-pressing process. The addition of LT powders promotes the resin flow during hot pressing and makes the surfaces of insulation layers flat and smooth. Figure 6c depicts the internal structures of LT-CCLs. The resin and ceramic powders are firmly anchored to the E-glass fibers, which improve the mechanical strength of CCLs. The cross-sectional micrographs of LT-CCLs are shown in Figure 6f. It is apparent that the brighter area is copper foil, which is confirmed in the corresponding element mapping of this area (Figure 7). The SEM mapping also shows that the LT ceramic powders are homogeneously distributed in the insulation layer of LT-CCLs, which can reduce the dielectric loss and water absorption.

### 3.2. Dielectric Properties

**Figure 8:**
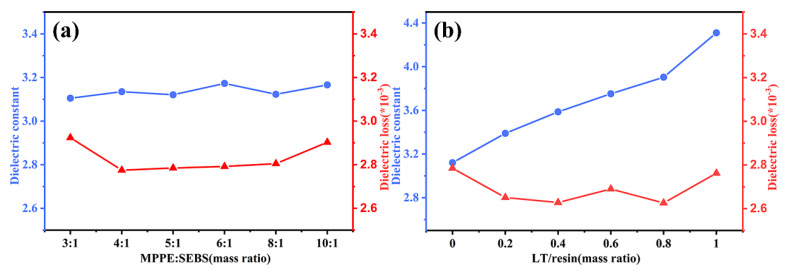
Dielectric constant and dissipation factors of (**a**) P-CCLs and (**b**) LT-CCLs.

The dielectric properties of composite laminates mainly depend on the polarizability of resin matrix and inorganic fillers. Besides, the porosity, defects, and interface between different components are also important and worth considering. The dielectric properties of P-CCLs with different weight ratio between MPPE and SEBS are shown in Figure 8a. The dielectric constant of P-CCLs maintains at 3.1. The polarizability of MPPE is close to SEBS and the resin matrix shows a very low Dk of about 2.5. The higher dielectric constant of E-glass fabrics (4.5) is the chief reason for the rise of dielectric constant of P-CCLs. The dielectric loss of P-CCLs shown in Figure 8a is within 0.0027–0.0029 at 10 GHz, which is a quite competitive value in the manufacturing and application of CCLs.

Figure 8b depicts the effect of LT ceramic powders content on the Dk and Df of LT-CCLs when the weight ratio of MPPE to SEBS is 5:1. The dielectric permittivity of LT powders is 22.3, which is larger than the dielectric constant of resin and E-glass fabrics. Therefore, the dielectric constant of LT-CCLs has a trend to increase with the addition of LT powders. With the increase of LT/resin, the dielectric constant rise steadily from 3.1 to 4.3. Meanwhile, the dielectric loss of composite laminates gets lower and remains between 0.0026 and 0.0028 at 10 GHz for the addition of LT ceramic powders. Besides, the dielectric loss of LT-CCLs exhibits a slight rise with the weight ratio increased to 1. Several common reasons may account for the rise of dielectric loss, that is the increasing defects and surfaces between resin and LT ceramic powders [15]. Nevertheless, these above results show a large decrease of dielectric loss in high-frequency CCLs.

### 3.3. Thermal Properties

**Figure 9:**
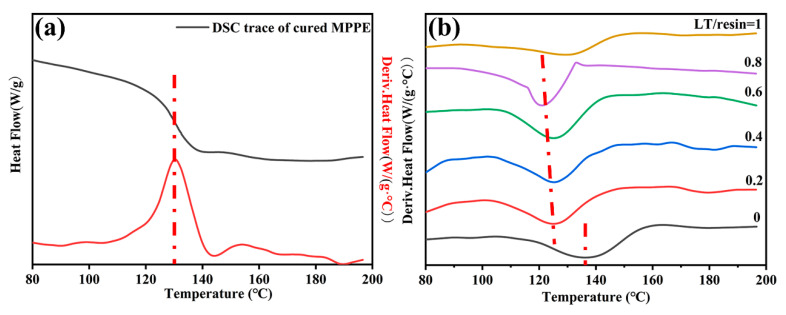
DSC curves of (**a**) cured MPPE and (**b**) LT-CCLs.

Glass transition temperature (Tg) reflects the maximum application temperatures, which is up to the movement capacity of molecular chains at certain temperature [7]. It is assumed that the Tg of crosslinked polymers is a function of the crosslink density [22,23]. Figure 9a depicts the DSC curves of the cured MPPE in the temperature range of 80–200 °C. Combining with suitable computer software, the glass transition temperature (Tg) of MPPE is around 130 °C. Meanwhile, since DTBP was used as initiator and no other crosslinking agents were added, the Tg of CCLs maintain between 123 and 137 °C. The addition of SEBS slightly increased the Tg of resin from 130 °C to 137 °C, indicating that a small amount of SEBS can help to restrain the movement of molecular chains. Figure 9b also demonstrates that the submicron-sized LT can slightly lower the Tg of CCLs, which means the crosslinking density reduces with the addition of LT powders. Several hypotheses have been put forward to explain the Tg drop, such as the plasticizing effect of residual organics on the particles [24], the reduced crosslink density of resin matrix [25] or the inhomogeneous distribution of inorganic fillers [26]. Moreover, the DSC trace of the cured MPPE displays no obvious exothermic peak, which reflects the curing reaction is completed.

For CCLs, the *z*-axis coefficient of thermal expansion (CTE) is of crucial importance. The CTE of copper foils is about 18 ppm/°C. The stresses can be generated between the interface of the copper foil and the composite laminates, causing the delamination of CCLs. The CTE of LT-CCLs below the Tg was tested from 20 °C to 100 °C and are shown in Table 1. The CTE value of P-CCLs without LT powders added is 80 ppm/°C. With the increase of LT ceramic powders, the lowest CTE (40 ppm/°C) was obtained when the weight fraction is 0.6. The low CTE of Li_2_TiO_3_ ceramic powders and the constraint of mobility due to the interaction of LT powders and resin are considered to cause the reduced CTE of LT-CCLs [27,28,29].

### 3.4. Flexural Strength and Moisture Absorption

**Figure 10:**
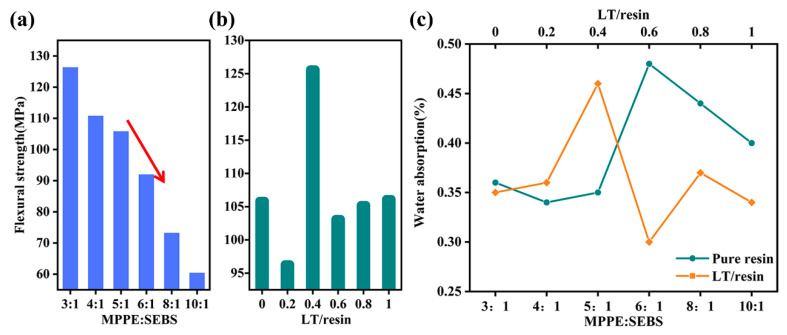
Flexural strength of (**a**) P-CCLs and (**b**) LT-CCLs; (**c**) water absorption of P-CCLs and LT-CCLs.

The flexure strength of CCLs is mainly determined by the crosslink density, porosity, and interface defects of the composite laminates [30]. Figure 10a shows the mean flexure strength of P-CCLs vary with the different weight ratios of MPPE and SEBS. SEBS is known as a toughening modifier. With the weight ratio change from 3:1 to 10:1, the mean flexure strength declines rapidly from 127 MPa to 60 MPa. However, too much SEBS will bring the glass transition temperature (Tg) of resin matrix down, which may restrict the application of MPPE in the CCLs. Additionally, the increase of SEBS can lead to a rapid rise in the viscosity of glue, creating difficulties for dipping fabrics production process. Based on these results, the suitable ratio of SEBS to MPPE is 20 wt %. Meanwhile, P-CCLs show relatively high Tg value, high flexure strength, and dielectric properties in this proportion.

Figure 10b shows the mean flexure strength of LT-CCLs with increasing weight ratios of LT/resin. With the weight ratio of LT/resin increasing from 0 to 1, the flexural strength of LT-CCLs increases first and then declines. The increase of flexure strength can be attributed to the debonding of the filler–polymer interface and the “nail-anchor” effect between LT ceramic powders and resin matrix, which rises the resistance of crack expansion and the toughness of LT-CCLs [31]. However, the addition of LT filler would reduce the crosslink density of resin matrix, which is showed in DSC curves of LT-CCLs (Figure 9). Despite E-glass fabrics and LT filler were modified by PhTES and KH570 separately, the increasing content of LT filler still causes the increase of interface defects, which decreases the flexure strength of composite laminates. These factors influence the flexure strength collectively and exhibited the highest flexural strength at the ratio of 0.4 [17].

Water absorption is another crucial parameter to CCLs, since the water can tremendously increase the dielectric loss. Water can be absorbed in the interface of ceramic powders and resin, and the interfacial polarization causes the extraordinary rise of Dk and Df [32]. The water absorptions of LT-CCLs shown in Figure 10c are in the range of 0.3%–0.5% and nearly as low as the P-CCLs. The reason can be found from the SEM pictures of CCLs’ insulation layers (Figure 6) and FTIR spectra of LT powders before and after modification (Figure 5). The addition of LT powders promotes the resin flowing and makes the surfaces of insulation layers flat and smooth. Meanwhile, the organic functional groups of KH570 and PhTES improve the hydrophobicity of LT powder and E-glass fabrics. Consequently, the water absorption of CCLs in this experiment can be attributed to the surface microcrack of insulation layers of CCLs.

MPPE/SEBS filled with LT ceramic powders exhibit superior properties compared to other composite laminates (Table 2). The obtained CCLs maintain low Dk (3.6) as well as low Df (0.0026) at high frequency and show relatively high flexural strengths (125 MPa) without adding crosslinking agents. These results indicate that CCLs with MPPE/SEBS and LT ceramic have great application prospect.

## 4. Conclusions

In summary, modified polyphenylene ether (MPPE)-based resin was toughened by styrene-ethylene/butylene-styrene (SEBS) and the as-prepared composite laminates show excellent dielectric properties with Dk = 3.1, Df < 0.003 at 10 GHz. When the weight ratio was MPPE: SEBS = 5:1, CCLs showed relatively high Tg (137 °C) and high flexure strength (105 MPa) without the addition of crosslinking agent. Furthermore, Li_2_TiO_3_ ceramic (LT) powders with excellent dielectric properties were fabricated by solid state sintered technology and added into the resin matrix. The E-glass fabrics and synthesized LT particles were modified by Phenyltriethoxysilane (PhTES) and KH570 respectively to improve the interfacial compatibility. The composite laminates showed increasing Dk, lower dielectric loss and decreased CTE with the addition of LT ceramic powders. Meanwhile, all composite laminates show low moisture absorption of 0.3%–0.5%. These results demonstrate that MPPE/SEBS filled with Li_2_TiO_3_ has strong competitiveness in the field of high-frequency copper clad laminates.

## Figures and Tables

**Table 1:** The coefficient of thermal expansions of LT-CCLs.

LT/Resin (Mass Ratio)	0	0.2	0.4	0.6	0.8	1
Relative density (g/cm^−3^)	1.51	1.60	1.66	1.71	1.76	1.78
CTE(ppm/℃)	80	75	73	40	-	76

**Table 2:** Comparison of properties of polymeric composites.

Composites	Loading	Dk	Df	F(Hz)	Flexural Strengths (MPa)	Reference
MgSiO_3_-SiO_2_-hBN/CE	50 vol%	6.8	0.03	10^3^–10^6^	─	[6]
HST/CE	0.7 wt.%	2.6	0.01	10^6^	145	[7]
rPPE/EP	─	3.76	0.0021	10^7^	─	[9]
BMI/glass fabrics	─	3.57	0.0053	10^6^	458.3	[33]
BN/PPE	48 wt.%	3.94	0.004	5 × 10^9^	300	[15]
MPPE/LT/glass fabrics	LT/MPPE = 0.4	3.6	0.0026	10^10^	125	This work
